# Atherosclerotic Cell Fates: A Single-Cell View of ER Stress

**DOI:** 10.3390/jcdd13090435

**Published:** 2026-09-03

**Authors:** Shuyi Li, Xiang Lu

**Affiliations:** Geriatrics, Sir Run Run Hospital, Nanjing Medical University, Nanjing 211000, China

**Keywords:** atherosclerosis, ER stress, scRNA-seq, macrophage

## Abstract

The role of endoplasmic reticulum (ER) stress in atherosclerosis has long been recognized, but whether it acts as a uniform pathological signal across all cell types has remained unclear. With the advancement of single-cell sequencing technology (scRNA-seq), we can analyze this problem at the resolution of a single cell subpopulation. In this review, we have synthesized the recent evidence from single-cell studies and proposed the “cell type-specific differential response” mode. We believe that endoplasmic reticulum stress does not universally have a pro-atherosclerotic effect. On the contrary, it shows a differential pattern in different atherosclerotic-related cell subpopulations. For example, endoplasmic reticulum stress is activated in monocytes but inhibited in certain macrophage subsets and endothelial cell subsets and dynamically regulated during phenotypic transitions in smooth muscle cells. We explored the underlying mechanisms of this heterogeneity, its impact on plaque progression, and the theoretical basis of subpopulation targeted treatment strategies. Ultimately, we concluded that recognizing the cellular heterogeneity of endoplasmic reticulum stress is the foundation for understanding and precisely intervening in atherosclerotic diseases.

## 1. Introduction

Atherosclerosis (AS) and its complications are among the leading causes of death worldwide [1,2]. This chronic inflammatory disease is characterized by the formation of lipid plaques within the arterial wall [3]. The pathogenesis involves complex interactions among macrophages, smooth muscle cells (SMCs), endothelial cells, and various immune cell subsets. These cells all participate in the formation, progression, and eventual rupture of the plaques. Although lipid-lowering and anti-inflammatory therapies have made significant progress, many patients still experience adverse cardiovascular events, underscoring the need for a deeper understanding of the cellular and molecular mechanisms driving AS.

The endoplasmic reticulum (ER) is a core cellular organelle responsible for protein folding, lipid synthesis, and calcium homeostasis. When the protein folding capacity of the endoplasmic reticulum is overloaded due to stress factors such as oxidative stress, lipid accumulation, hypoxia or inflammatory cytokines, misfolded proteins accumulate in the lumen of the endoplasmic reticulum, thereby triggering the unfolded protein response (UPR). This state is known as “endoplasmic reticulum stress” [4]. The unfolded protein response (UPR) functions through three classic pathways, namely the IRE1α-XBP1 pathway, the PERK-eIF2 α-ATF4 pathway, and the ATF6 pathway. Under acute or mild stress conditions, UPR restores endoplasmic reticulum (ER) homeostasis by inhibiting protein synthesis, upregulating the expression of molecular chaperones, and enhancing ER-associated degradation (ERAD). However, in chronic or severe stress situations, UPR shifts to pro-apoptotic signaling, activating CHOP and other downstream effectors, promoting cell death and tissue damage [5]. In recent years, endoplasmic reticulum stress has been confirmed to be closely related to various cardiovascular diseases, including atherosclerosis, myocardial hypertrophy and heart failure, etc. [6], and thus has become a current research hotspot.

Traditional research on endoplasmic reticulum stress mainly relies on bulk tissue analysis or in vitro experiments of single-cell types, which are difficult to fully reveal the complexity of the plaque microenvironment. The emergence of single-cell RNA sequencing (scRNA-seq) has changed this situation. The recent emergence of single-cell RNA sequencing (scRNA-seq) has revolutionized this field by enabling transcriptomic profiling at the resolution of individual cells. It can perform transcriptome analysis at the resolution of individual cells, resolve cellular heterogeneity, identify rare cell populations and reconstruct differentiation trajectories [7]. In the study of atherosclerosis, this technique has identified macrophage subsets with diverse functions, such as Lyve1^+^ tissue-resident macrophages, Il1b^+^ inflammatory macrophages, and Trem2^+^ foam cell-like macrophages. These subpopulations could not be distinguished by traditional methods before [8]. ScRNA-seq has also revealed unexpected phenotypic changes in smooth muscle cells (SMCs), including transformations into macrophage-like and fibroblast-like states [9], and identified multiple endothelial cell subpopulations with unique functional characteristics [10]. These discoveries have opened new avenues for understanding the cellular ecosystem of AS and have raised a critical question: how does ER stress, a ubiquitous cellular stress response, differentially impact these newly identified cell subsets?

While previous reviews have discussed ER stress in atherosclerosis, most studies have focused on a single cell type or a single UPR pathway. Currently, there is a lack of a synthesis that explains how ER stress differentially regulates multiple cell types from this single-cell perspective. In this review, we synthesize recent single-cell evidence to propose a “cell type-specific differential response” model of ER stress in atherosclerosis. We argue that ER stress is not a monolithic pro-atherogenic signal. Rather, its direction and outcome, whether activation or suppression, inflammation or resolution, phenotypic switching or quiescence, depend critically on the specific cell subset and its pre-existing transcriptional state. Specifically, we highlight three core pillars of this model: (1) ER stress exhibits divergent patterns across cell subsets, elevated in monocytes, suppressed in certain macrophage and endothelial subsets, and subject to dynamic, context-dependent regulation in SMCs; (2) the functional output of the same UPR pathway (e.g., PERK-CHOP) varies across cell types depending on the cellular context and stress duration; and (3) therapeutic targeting of ER stress must consider this heterogeneity, moving beyond global activation or inhibition toward subset-specific modulation.

We begin by outlining the core UPR pathways, followed by a systematic analysis of ER stress regulation in macrophages, SMCs, and endothelial cells, with particular emphasis on the subset-specific mechanisms revealed by scRNA-seq, and conclude by discussing therapeutic implications and proposing testable hypotheses for future research.

## 2. The Core Signaling Pathway of ER Stress

**Figure 1:**
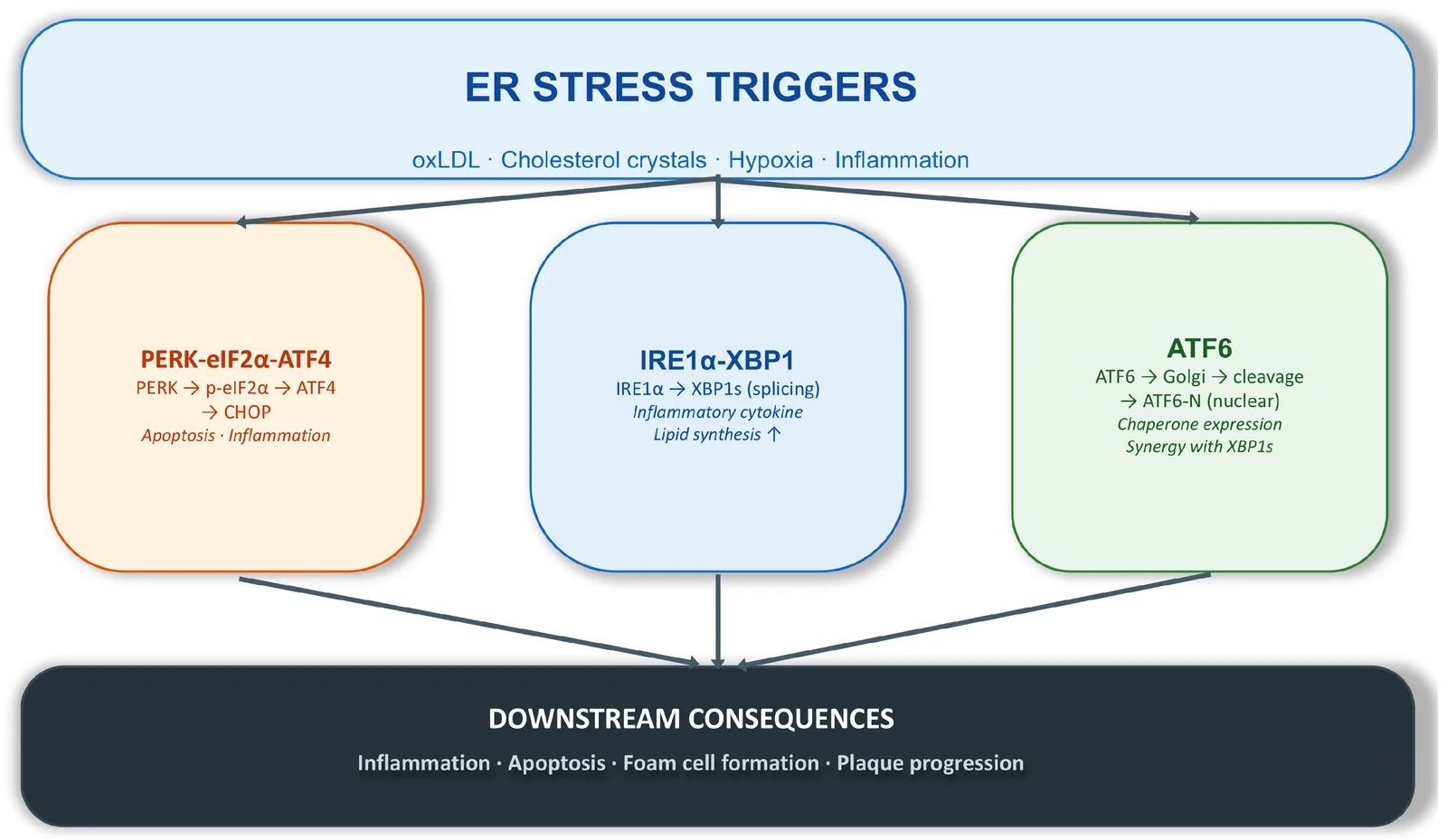
Schematic overview of the three major ER stress signaling pathways. ER stress is triggered by various atherogenic stimuli, including oxidized LDL (oxLDL), cholesterol crystals, hypoxia, and calcium dysregulation. The unfolded protein response (UPR) is transduced through three canonical branches: IRE1α-XBP1, PERK-eIF2α-ATF4, and ATF6. The IRE1α-XBP1 pathway promotes inflammatory cytokine production and lipid synthesis. Upward arrows denote increased expression or activation. The PERK-eIF2α-ATF4 pathway primarily mediates apoptosis via CHOP upregulation. The ATF6 pathway induces chaperone expression and synergizes with XBP1s. These pathways converge on downstream effects including inflammation, apoptosis, and foam cell formation, collectively contributing to plaque progression and instability.

ER stress has three main pathways (Figure 1), such as the IRE1α-XBP1 pathway, the PERK-eIF2α-ATF4 pathway, and the ATF6 pathway. The IRE1α-XBP1 pathway is that IRE1α cuts XBP1 mRNA to generate the active transcription factor XBP1s, promoting the expression of lipid synthesis genes, such as SREBP-1c, and inflammatory factors (IL-6, IL-8). This pathway can inhibit the cholesterol efflux protein (such as ABCA1), simultaneously exacerbating the formation of foam cells. The second pathway is the PERK-eIF2α-ATF4 pathway. PERK phosphorylates eIF2α to inhibit translation but activates ATF4, upregulating CHOP (pro-apoptotic factor) and inflammation-related genes. Then CHOP promotes macrophage apoptosis, leaking out their lipid contents and leading to the formation of the necrotic cores. The last pathway is the ATF6 pathway. Following its translocation to the Golgi apparatus and cleavage, ATF6 activates genes involved in lipid metabolism (such as LDLR) and inflammation and synergizes with XBP1s to amplify the unfolded protein response (UPR) [4].

## 3. Cellular Heterogeneity of Atherosclerosis from a Single-Cell Perspective

To provide a concise overview of the cell type-specific ER stress responses discussed in this review, we summarize their key features in Table 1.

**Figure 2:**
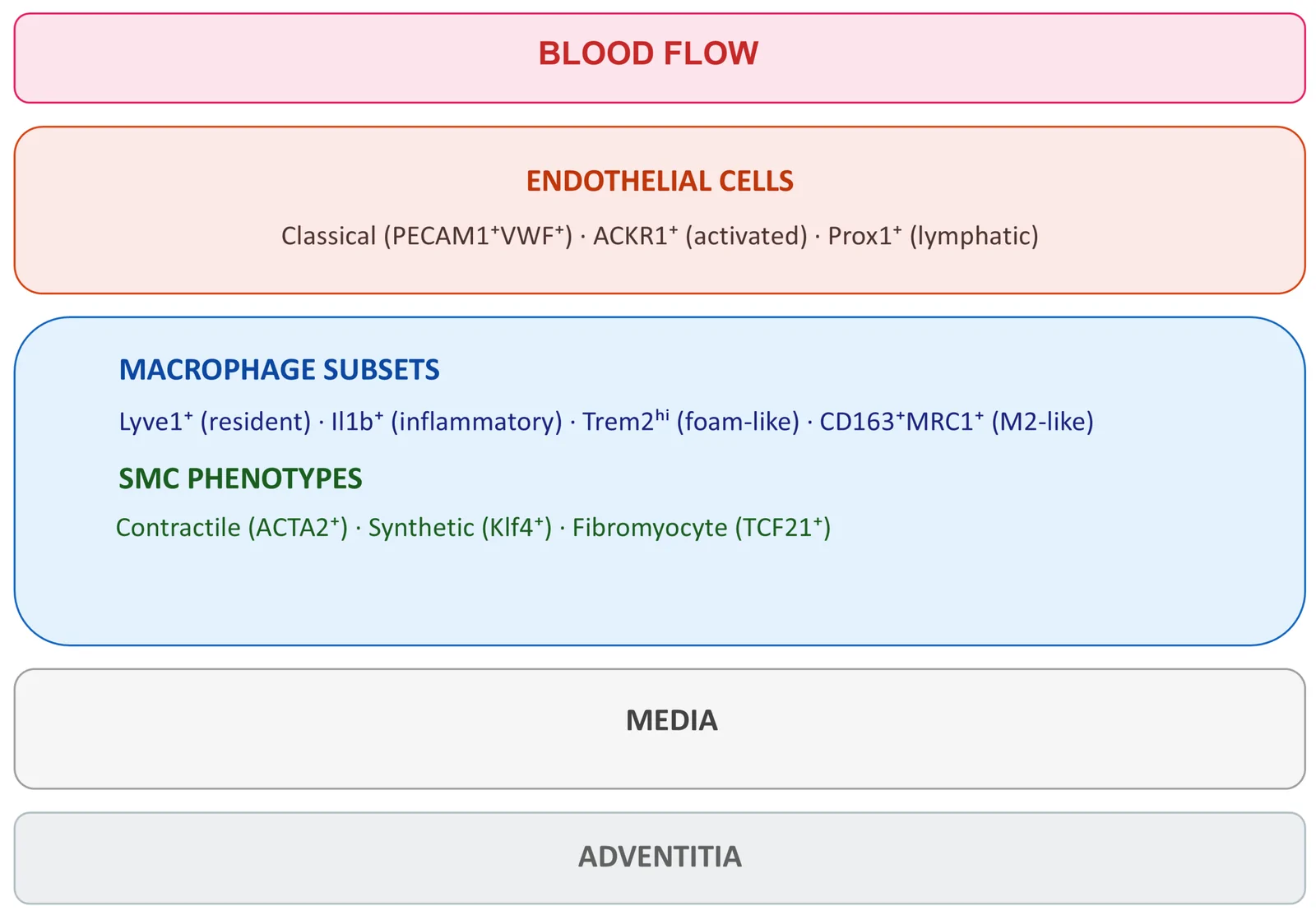
Cellular heterogeneity in atherosclerotic plaques revealed by single-cell RNA sequencing. ScRNA-seq has identified diverse cell subsets within atherosclerotic lesions. Macrophage subsets include Lyve1^+^ tissue-resident macrophages (anti-inflammatory), Il1b^+^ inflammatory macrophages (pro-inflammatory), and Trem2^hi^ foam cell-like macrophages (lipid-laden). Smooth muscle cells (SMCs) exhibit phenotypic switching from contractile (ACTA2^+^TAGLN^+^) to synthetic, macrophage-like, and osteogenic-like states, regulated by transcription factors Klf4 and TCF21. Endothelial cells comprise subsets including lymphatic (Prox1^+^Lyve1^+^), matrix-regulating, and ACKR1^+^ activated endothelial cells. This cellular heterogeneity provides the foundation for understanding cell type-specific ER stress responses.

Multiple cell types coexist within AS lesions and display high functional heterogeneity (Figure 2). The application of scRNA-seq has significantly deepened our understanding of this complexity, revealing diverse immune, stromal, and vascular wall cell populations. Here, we focus on three major cell types most relevant to ER stress, macrophages, smooth muscle cells, and endothelial cells, and summarize their subset heterogeneity uncovered by recent single-cell studies.

### 3.1. Macrophage Subpopulations and Their Functions

Macrophages are the most abundant immune cells in AS plaques, and their functional state directly influences plaque inflammation, expression levels, and stability. ScRNA-seq studies have identified three functionally distinct macrophage subsets in atherosclerotic plaques. Lyve1^+^ tissue-resident macrophages highly express Lyve1, Folr2, and Mrc1, and exhibit anti-inflammatory and tissue-repair functions similar to M2-type macrophages [7,11]. In early AS, these cells may play a protective role. Il1b^+^ inflammatory macrophages highly express pro-inflammatory factors such as IL-1β, TNF-α, and CCL3, resemble M1-type macrophages, and promote plaque inflammation and instability [8]. Trem2^+^ foam-like macrophages highly express Trem2, SPP1, and CD9, and are closely associated with lipid metabolism, oxidative stress, and foam cell formation [12]. These cells are enriched in advanced plaques and may contribute to plaque calcification and necrosis. Other subpopulations include Proliferative macrophages expressing GATA2 [13], FCGR1A^+^ inflammatory macrophages [8], and others. These subpopulations undergo dynamic changes during the progression of AS and are regulated by microenvironmental signals. Endoplasmic reticulum (ER) stress plays a crucial role in this process. It regulates the polarization, apoptosis and lipid metabolism of macrophages, thereby shaping their functional phenotypes. Regarding the different sensitivities of these macrophage subpopulations to ER stress and the potential mechanisms underlying their differential responses, we will discuss them in Section 4.

### 3.2. Phenotypic Transformation of Smooth Muscle Cells

Vascular smooth muscle cells (SMCs) show remarkable plasticity in AS. They can switch from a contractile phenotype to synthetic, macrophage-like, fibroblast-like, or even osteoblast-like phenotypes; this process is known as SMC phenotypic transformation. Multiple factors, including cholesterol, inflammatory cytokines, and oxidative stress, regulate this process. Studies have shown that cholesterol-induced UPR activates the transcription factor Klf4, which serves as a master regulator of SMC phenotypic switching by promoting macrophage- and fibroblast-associated gene expression while suppressing contractile genes [14,15]. CCN2 (cellular communication network factor 2), on the other hand, maintains the vascular protective function of SMCs by inhibiting UPR-related genes and reducing lipid accumulation [16]. Furthermore, integrative single-cell analysis has revealed that SMCs can transition to a fibromyocyte/fibrochondrocyte phenotype, which is associated with the fibrous cap and calcified regions of plaques [17]. SMC phenotypic transformation not only affects plaque stability but also exacerbates AS progression through the generation of foam cells and pro-inflammatory mediators. The role of ER stress in driving SMC phenotypic switching, and its implications for plaque stability, will be discussed in Section 5.

### 3.3. Endothelial Cell Dysfunction and Subpopulation Differentiation

Endothelial cells line the inner surface of blood vessels and play a critical role in maintaining vascular homeostasis. ScRNA-seq studies have revealed that endothelial cells are not a homogeneous population but consist of multiple subpopulations with distinct transcriptomic features. These include lymphatic endothelial cells (Prox1^+^Lyve1^+^), matrix-regulating endothelial cells characterized by high expression of extracellular matrix genes, lipid metabolism-related endothelial cells, and ACKR1^+^ endothelial cells, which may be involved in chemokine scavenging and immune cell trafficking [10,18]. However, compared with macrophages and SMCs, the functional implications of these endothelial subsets in AS, particularly their differential responses to ER stress, remain poorly understood and warrant further investigation. Although the potential differential response of these endothelial cell subsets to ER stress is currently undefined, we explore it in Section 6.

## 4. Differential ER Stress Responses in Macrophage Subsets

### 4.1. Macrophage Subsets and ER Stress: An Overview

As discussed in Section 3.1, multiple functionally distinct macrophage subsets have been identified in atherosclerotic plaques. A key question is whether ER stress acts equally or exerts a differential effect across these subgroups. This chapter discusses the monocyte and macrophage subset differences in the endoplasmic reticulum stress patterns and their significance to the pathogenesis of atherosclerosis (AS).

### 4.2. Monocytes: ER Stress Activation Drives Pro-Inflammatory Differentiation

Monocytes are the precursor cells of macrophages and are also the immune cells that exhibit the most significant activation response. When circulating monocytes enter the plaque microenvironment, they are exposed to stress factors such as oxidized lipids, hypoxia, and inflammatory cytokines. These factors can rapidly trigger the unfolded protein response (UPR) [7,19]. This activation is not a transient event. Monocytes still maintain a relatively high level of endoplasmic reticulum stress in advanced atherosclerotic plaques. This may indicate the existence of a persistent pro-inflammatory driving mechanism throughout the disease process [19,20].

The pro-inflammatory effects mediated by ER stress in monocytes mainly occur through the PERK-eIF2α-ATF4 pathway and the IRE1α-XBP1 pathway. The PERK pathway upregulates the proinflammatory cytokines IL-1β and TNF-α through ATF4 [21], while the IRE1α pathway enhances inflammatory signaling through the activation of JNK and NF-κB [22]. These two pathways work together to keep monocytes in a pro-inflammatory state. Meanwhile, ER stress-induced CHOP activation can mediate pyroptosis in THP-1 monocytes through the NLRP3 inflammasome [23], establishing a vicious cycle of “inflammation–death–more inflammation.”

Monocytes, as the main source of macrophages, their high endoplasmic reticulum stress state may lay the foundation for the subsequent functional differentiation of macrophage subsets and explain why some macrophages retain relatively high stress characteristics. However, this activation phenomenon is not common in all macrophage subpopulations.

### 4.3. Trem2^hi^ Macrophages: ER Stress and Foam Cell Formation

Among the multiple differentiation trajectories of monocytes, Trem2^hi^ macrophages are one of the most representative subsets regulated by ER stress in advanced plaques. These cells are particularly abundant in advanced plaques, with higher expression levels of Trem2, SPP1 and CD9, and are closely related to lipid metabolism, oxidative stress and foam cell formation [24,25]. In these cells, ER stress promotes lipid accumulation and foam cell formation through the following three ways.

The first is to inhibit cholesterol efflux. Endoplasmic reticulum stress significantly downregulates the cholesterol efflux transporters ABCA1 and ABCG1 through the CHOP protein [11] and also inhibits the SR-B1 protein [11], thereby hindering the clearance of excess lipids. The second approach is to enhance lipid uptake. ER stress upregulates scavenger receptors SR-A and CD36 [11], increasing the lipid influx. Finally, ER stress interferes with the lysosome acidification process and autophagosome maturation, preventing macrophages from effectively degrading the ingested lipids, and these accumulated cholesterol crystals further exacerbate the inflammatory response [26].

These three methods collectively form a self-reinforcing vicious cycle. Lipid accumulation induces endoplasmic reticulum stress, which in turn inhibits cholesterol efflux, promotes lipid uptake and impairs lysosomal function, ultimately accelerating foam cell formation and necrotic core expansion. The IRE1α-XBP1 pathway plays a central role in this process. Activated IRE1α kinase can directly phosphorylate FMRP, inhibit cholesterol excretion, and enhance the phagocytic function of macrophages [27], while inhibiting IRE1 can alleviate atherosclerotic lesions [27].

The formation of foam cells in Trem2^hi^ macrophages induced by ER stress further confirms that ER stress has a pro-atherosclerotic effect in specific cell subpopulations.

### 4.4. Reduced ER Stress in M2-like Macrophages: An Overlooked Phenomenon

In contrast to the significant endoplasmic reticulum stress activation phenomenon in TREM2-activated macrophages, M2-like macrophages exhibit distinct characteristics. It is traditionally believed that M2-like macrophages are the main group of anti-inflammatory and tissue repair cells. Due to the high expression of ER molecular chaperones, M2-like macrophages have stronger ER stress tolerance [25,28], which is considered to be the basis for their survival and repair function in the chronic inflammatory microenvironment.

However, M2-like macrophages in the atherosclerotic microenvironment may not conform to this paradigm. GRP78 (HSPA5), as a key endoplasmic reticulum chaperone protein and the main regulatory factor for M2 polarization, is crucial for the differentiation of M2-type macrophages. Its downregulation inhibits M2 polarization and promotes M1-type differentiation through the JAK/STAT signaling pathway [29]. Multiple studies further indicate that the number of GRP78-positive cells in atherosclerotic plaques gradually decreases over time [30], and HSPA5 is generally downregulated in macrophages under atherosclerotic conditions [31]. These findings suggest that the function of the chaperone protein supporting the tolerance of M2-type macrophages to endoplasmic reticulum stress may be defective in the atherosclerotic microenvironment.

Based on these results, we have discovered a long-neglected possibility. In advanced plaques, M2-like macrophages may have lost their traditional advantage of endoplasmic reticulum stress tolerance and have entered a state of “insufficient molecular chaperone function”. But whether this state represents an adaptive response or the beginning of functional failure is unclear. Nevertheless, even so, this possibility challenges the long-held assumption and indicates that researchers need to re-evaluate the functional roles of these cells and their ability to respond to endoplasmic reticulum stress based on the specific circumstances of different cell subtypes.

The low endoplasmic reticulum stress level of M2-like macrophages in atherosclerosis suggests that endoplasmic reticulum stress shows differentiated responses among different macrophage subsets, rather than functioning as a unified pro-atherosclerotic signal.

### 4.5. Dynamic Regulation of ER Stress in Macrophages: A Pseudotime Perspective

Whereas the preceding sections examined differences in ER stress across subsets, pseudotime analysis offers a complementary perspective by ordering cells along a transcriptional continuum, allowing us to infer dynamic changes in ER stress during monocyte-to-macrophage differentiation. Single-cell RNA sequencing (scRNA-seq) can infer the dynamic trajectory of cell state transitions from static transcriptome data [32]. In the myeloid cells within atherosclerotic plaques, pseudo-time analysis revealed the path of differentiation from monocytes to different macrophage subpopulations, indicating the gradual loss of monocyte lineage genes such as Plac8 and Ifitm6, while genes related to foam cell macrophages were enriched along the differentiation trajectory [33,34].

Along these differentiation trajectories, the endoplasmic reticulum stress activity shows a distinct but non-monotonic change pattern. During the early stage of differentiation, when monocytes transform into macrophages, the activity of endoplasmic reticulum stress briefly increases, while in the terminal macrophage subpopulation, it drops to a lower level [33]. This fluctuating dynamic change indicates that the brief activation of endoplasmic reticulum stress may accompany the initiation of the differentiation process and can serve as a permission signal to promote subsequent metabolism and transcriptional reprogramming.

It is worth noting that the baseline level of endoplasmic reticulum stress varies across different differentiation stages. M2-like macrophages exhibit a relatively lower level, while SPP1^+^ macrophages retain higher stress characteristics [35]. This difference indicates that the microenvironment differentially regulates endoplasmic reticulum stress at different differentiation stages. The comprehensive trajectory analysis further reveals that certain genes such as HLA-B are dynamically regulated during VSMC differentiation, while ITLN1^+^ foam cells represent an end-stage differentiation state, characterized by enhanced lipid synthesis and upregulated gene expression related to the progression of AS [36,37]. We speculate that ER stress plays a “priming” role during early differentiation, while its subsequent decline may facilitate the maintenance of specific macrophage functions.

The pseudotime perspective thus reveals the dynamic evolution of ER stress along the differentiation axis, the “rise-and-fall” pattern, together with endpoint differences across subsets, jointly outlines a complete picture of differential ER stress responses within the macrophage lineage.

### 4.6. Chapter 4 Summary

This chapter examined the differential ER stress responses across the macrophage lineage, starting from monocytes. ER stress is significantly activated in monocytes and Trem2^hi^ macrophages, driving pro-inflammatory differentiation and foam cell formation. In M2-like macrophages, however, ER stress is reduced, potentially reflecting a state of chaperone insufficiency. Pseudotime analysis further revealed a “rise-and-fall” dynamic pattern of ER stress during monocyte-to-macrophage differentiation. In conclusion, these observations indicate that endoplasmic reticulum stress in macrophages is not a uniform pro-atherosclerotic signal. The direction of action and the final effect vary according to cell subsets and differentiation stages. This conclusion lays the foundation for exploring the difference in ER stress response between smooth muscle cells and endothelial cells in the following chapters.

## 5. Differential ER Stress Responses in Smooth Muscle Cells

### 5.1. Smooth Muscle Cell Plasticity and ER Stress: An Overview

The previous text has already elaborated that vascular smooth muscle cells (SMCs) possess high plasticity and can transform into various phenotypes. This chapter will explore the role of endoplasmic reticulum stress in driving this phenotypic plasticity. Unlike the differentiation pattern observed in macrophages, endoplasmic reticulum stress plays a more complex role in SMCs. It can not only promote the transformation of SMCs into pro-inflammatory and osteogenic phenotypes but also maintain their homeostasis balance under specific conditions. This chapter will focus on how ER stress drives the phenotypic switch of SMCs, promotes vascular calcification, and has a dual effect on the fate of SMCs.

### 5.2. ER Stress Drives SMC-to-Macrophage-like/Fibroblast-like Phenotypic Switching

The discovery of the trans-differentiation of smooth muscle cells (SMCs) into other cell types has challenged the traditional view of the pathogenesis of atherosclerosis (AS). Lineage tracing studies have confirmed that up to 30% of macrophage-like cells in AS lesions actually originate from SMCs [38]. Single-cell RNA sequencing further revealed that in the in vivo environment, SMCs mainly transformed into a unique fibroblast-like cell, “fibromyocytes”, rather than the typical macrophage phenotype [9]. The key trigger for this transdifferentiation is the activation of cholesterol-induced unfolded protein response (UPR). Cholesterol load can activate the PERK-eIF2α-ATF4 and IRE1α-XBP1 signaling pathways, thereby upregulating the expression of the transcription factor Klf4 [14,15]. Klf4 is the main regulatory factor for the phenotypic transformation of SMCs. It directly activates macrophage-related genes, such as CD68, Lgals3, and fibroblast-related genes, while inhibiting the expression of contraction-related genes such as ACTA2, TAGLN, and MYH11 through epigenetic mechanisms [14,15]. Persistent Klf4 expression drives SMCs to progressively lose their contractile function and acquire macrophage-like inflammatory and phagocytic features [15,38].

TCF21, a transcription factor highly expressed in SMCs and a causal gene for CAD, plays a critical regulatory role in SMC phenotypic modulation. SMC-specific knockout of TCF21 in mice markedly inhibited SMC phenotypic switching, reducing fibromyocyte numbers within lesions and impairing fibrous cap formation [9,39]. This suggests that TCF21-driven fibromyocyte transition contributes to fibrous cap stability rather than pathological transformation. Consistently, higher TCF21 expression in diseased human coronary arteries is associated with reduced CAD risk [9,39]. Integrative analysis of 22 scRNA-seq libraries (118,578 cells) further confirmed that SMC-derived fibromyocyte and fibrochondrogenic phenotypes are significantly enriched in fibrous cap and calcified regions, and are associated with CAD, myocardial infarction, and coronary calcification [17,39]. This study also identified LTBP1 and CRTAC1 as markers of these SMC-derived phenotypes, serving as proxies of atherosclerosis progression [39,40].

CCN2 (cellular communication network factor 2) also plays an important regulatory role in SMC phenotypic switching. Single-cell sequencing analysis has revealed that CCN2-deficient SMCs exhibit aberrant activation of ER stress-related pathways, endocytosis and lipid accumulation pathways, and key regulators of macrophage-like transdifferentiation [16]. CCN2 maintains vascular stability by inhibiting SMC-to-pro-inflammatory macrophage-like phenotype switching and reducing lipid accumulation and foam cell formation [16].

ER stress-driven SMC-to-fibroblast-like (fibromyocyte) and macrophage-like phenotypic switching endows SMCs with pro-inflammatory and foam cell-forming capacity, representing one major “pro-atherogenic” output of ER stress in SMCs.

### 5.3. ER Stress Promotes SMC Osteogenic-like/Chondrogenic Transformation and Vascular Calcification

Vascular calcification is a hallmark of advanced atherosclerosis and a major contributor to plaque instability. SMCs are the primary source of osteogenic-like and chondrocyte-like cells in vascular calcification, and ER stress plays a critical driving role in this transdifferentiation process.

The PERK-eIF2α-ATF4 pathway is the central axis through which ER stress drives SMC osteogenic transformation. Studies have demonstrated that C5a-C5aR1 signaling activates the PERK-eIF2α-ATF4-CHOP pathway, upregulates ATF4 expression, and subsequently promotes the transcription of downstream osteogenic markers (OCN, IBSP), ultimately driving SMC-to-osteogenic-like transdifferentiation [41]. ATF4 further regulates CREB3L1, a transcription factor involved in bone development, which upregulates COL1α1 expression and accelerates calcification. Experimental evidence confirms that ER stress inhibition, for example, using 4-PBA, or ATF4 knockdown, blocks CREB3L1-mediated pro-calcification effects [41].

In addition to osteogenic transformation, SMCs can also transition to chondromyocytes (chondrocyte-like cells). The environment-sensing aryl hydrocarbon receptor (AHR) has been shown to inhibit the chondrogenic fate of SMCs [42], and changes in AHR expression during AS may influence the tendency of SMCs toward chondrogenic phenotypic switching [42]. TXNIP (thioredoxin-interacting protein) similarly suppresses SMC osteogenic/chondrogenic switching, and its downregulation in AS may relieve this protective inhibition [43]. Damaged SMCs undergo dedifferentiation into a synthetic phenotype under ER stress, subsequently transforming into osteoblast-like or chondrocyte-like cells, driving both medial and intimal calcification [44].

Under the dual effects of ER stress, which promotes both SMC apoptosis and calcification, SMC numbers decrease while residual SMCs adopt osteogenic/chondrogenic phenotypes, collectively weakening vascular wall stability and promoting necrotic core expansion [45]. This mechanistic chain tightly links ER stress, SMC apoptosis, and vascular calcification.

ER stress-driven SMC-to-osteogenic-like/chondrogenic transformation represents another “pro-atherogenic” output pathway of ER stress in SMCs, operating in parallel with fibromyocyte/macrophage-like transdifferentiation to accelerate plaque destabilization.

### 5.4. The Dual Role of ER Stress in SMCs: Pro-Apoptotic Versus Adaptive Protective Effects

It has been expounded in the previous text that endoplasmic reticulum stress has a “pro-atherosclerotic” effect in smooth muscle cells (SMCs), driving phenotypic transformation and vascular calcification. However, under certain conditions, moderate activation of the unfolded protein response (UPR) may exert protective functions.

Under severe or persistent endoplasmic reticulum stress conditions, the PERK-eIF2α-ATF4-CHOP signaling pathway is activated. It induces apoptosis in smooth muscle cells (SMC) by upregulating the pro-apoptotic protein BAX and inhibiting the anti-apoptotic protein BCL-2 [24,46]. Moreover, endoplasmic reticulum stress can specifically upregulate the expression of RIPK3 rather than activating the classical apoptotic pathway, thereby driving SMC to undergo necrotic apoptosis [45]. This kind of SMC death caused by endoplasmic reticulum stress not only directly reduces the number of SMCS in the fiber cap but also aggravates the local inflammatory response by releasing damaging-related molecular patterns.

But not all endoplasmic reticulum stress is detrimental. There is evidence suggesting that mild and continuous activation of the unfolded protein response (UPR) may be an adaptive response. As a non-splice isoform of XBP1 mRNA, XBP1u has been shown to maintain contractile homeostasis of smooth muscle cells by interacting with FoxO4 transcription factor [47]. Moreover, the cholesterol-induced phenotypic transformation of smooth muscle cells also depends on the activation of UPR [15], indicating that moderate UPR signal transduction may be a normal response of cells to environmental stress rather than a pathological signal. The transformation of smooth muscle cells into fibroblast-like cells mediated by TCF21 is also considered to have an arterial protective effect [9,39], suggesting that the phenotypic transformation of smooth muscle cells is not a simple degenerative process but an adaptive response in specific circumstances.

This indicates that differentiating between “pathological endoplasmic reticulum stress” and “adaptive unfolded protein response (UPR)” is crucial for understanding the fate determination of smooth muscle cells (SMC). When the intensity of endoplasmic reticulum stress exceeds a certain threshold or its duration is too long, the adaptive protective mechanism may transform into pro-apoptotic signal transduction. Unfortunately, it is still difficult to precisely define this threshold under in vivo conditions. And this dual effect adds another layer of complexity to the fate regulation of SMCs mediated by ER stress.

### 5.5. SMC Phenotypic Switching: A Pseudotime Perspective

Unlike the “rise-and-fall” dynamic pattern of ER stress along the macrophage differentiation trajectory, pseudotime analysis of SMCs reveals a more nuanced picture.

Single-cell pseudotime analysis has delineated continuous trajectories of SMC phenotypic switching across multiple models. In carotid atherosclerotic plaques, pseudotime analysis demonstrated a trajectory originating from contractile VSMCs, progressing through intermediate foam cell-like and inflammatory synthetic states, and ultimately transitioning into fibroblast-like phenotypes [39,40]. Byun et al. obtained approximately 23,661 cells from four carotid plaques and identified four transcriptionally distinct VSMC phenotypes [39]. In another study, Gao et al. classified VSMCs into six clusters, with clusters 4, 5, and 6 identified as contractile VSMCs and clusters 1, 2, and 3 as synthetic VSMCs [39,48], and identified “transitional cells” expressing both VSMC and fibroblast markers through pseudotime analysis [48]. Pan et al. further utilized dense time-course single-cell transcriptomic and epigenetic mapping to reveal complex SMC transitions toward fibromyocyte (FMC) and osteochondrogenic (chondromyocyte, CMC) phenotypes [39,40].

Notably, the vast majority of SMCs in advanced plaques exhibit a gradual increase in contractile phenotype scores and a concurrent decrease in synthetic phenotype scores, accompanied by a mild but sustained increase in ER stress scores [35]. This pattern suggests that these SMCs may not be undergoing de-differentiation but rather a reparative or re-differentiation phenotypic transition [40]. Gao et al. also showed that contractile VSMCs can reacquire contractile characteristics under specific conditions [48].

This phenomenon, in which mild ER stress elevation accompanies contractile phenotype recovery, appears paradoxical at first glance, as ER stress is typically viewed as a pro-dedifferentiation signal. However, considering the role of XBP1u in maintaining the contraction homeostasis of smooth muscle cells [47], it is possible that in advanced plaques, some smooth muscle cells may moderately upregulate the unfolded protein response (UPR), but not reaching the threshold for cell apoptosis, thereby supporting the re-synthesis of contractile proteins and facilitating a more stable phenotype transition. This adaptive UPR upregulation differs from pathological ER stress overactivation and represents a cell-autonomous homeostatic maintenance mechanism.

It must be emphasized, however, that this interpretation remains speculative at present. The scarcity of early-stage SMCs limits the ability of single-cell sequencing to capture the initiating events of phenotypic switching. The dynamic patterns described above await validation through lineage-tracing or in vitro differentiation experiments.

The pseudotime dynamics of ER stress in SMCs suggest that the direction of ER stress score changes (increase or decrease) cannot simply be labeled as “beneficial” or “detrimental”. The critical factor is the cellular state and stress conditions in which these changes occur.

### 5.6. Chapter 5 Summary

This chapter examined the differential responses of SMCs to ER stress. In SMCs, ER stress serves as both a driver of pro-atherogenic phenotypic changes (via Klf4 and ATF4-CREB3L1) and, under certain conditions, a protector of contractile homeostasis (via XBP1u and TCF21-mediated fibromyocyte transition). This duality, together with its intensity-dependent nature, distinguishes SMC responses from the subset-specific patterns seen in macrophages, and further illustrates the cell type-specificity of ER stress in atherosclerosis.

## 6. Differential ER Stress Responses in Endothelial Cells

### 6.1. Endothelial Dysfunction and ER Stress: An Overview

Endothelial cells form the innermost monolayer of the vascular wall, constituting the physical barrier between blood and the vessel wall. Endothelial dysfunction is considered the initiating event of atherosclerosis, characterized by reduced nitric oxide bioavailability, upregulated adhesion molecule expression, increased permeability, and acquisition of a pro-thrombotic phenotype.

Hemodynamic disorders, oxidative lipids and inflammatory cytokines can all induce endoplasmic reticulum stress in endothelial cells [49]. Endoplasmic reticulum stress activates NF-κB through the IRE1α and PERK pathways, promoting the expression of adhesion molecules and monocyte adhesion [49]. It also induces endothelial cell apoptosis through the upregulation of BAX mediated by CHOP and the inhibition of BCL-2 [46] and activates NADPH oxidase to cause the accumulation of reactive oxygen species (ROS) and uncoupling of endothelial nitric oxide synthase (eNOS), thereby exacerbating oxidative stress [46]. These three pathways work together to transform endothelial cells from the maintainers of vascular homeostasis into the initiating factors for plaque formation.

### 6.2. Differential ER Stress Features in Endothelial Subsets

Single-cell sequencing has revealed that endothelial cells are not a homogeneous population but consist of multiple functionally distinct subsets [10].

Classical endothelial cells highly express markers such as PECAM1, VWF, and CLDN5. In AS lesions, core ER stress genes (ERN1, XBP1, ATF6, HSPA5) in these cells are generally downregulated [50,51]. This observation appears paradoxical: if ER stress promotes dysfunction, why is ER stress reduced in endothelial cells in AS? One explanation is that in advanced AS, endothelial cells chronically exposed to extreme stress may actively downregulate UPR sensor expression through transcriptional reprogramming as a “stress adaptation” strategy to avoid chronic inflammation [52]. However, this adaptation may also render endothelial cells unable to mount a protective UPR upon future acute stress.

ACKR1^+^ endothelial cells are a subset highly expressing the atypical chemokine receptor 1, potentially involved in chemokine-driven inflammation and immune cell recruitment [18]. PLVAP^+^ endothelial cells highly express plasmalemma vesicle-associated protein, with increased expression suggesting enhanced endothelial permeability [53]. The ER stress features of these subsets in AS currently lack direct investigation, but their association with inflammation and permeability suggests they may exhibit ER stress regulation patterns distinct from classical endothelial cells.

In addition, scRNA-seq has identified lymphatic endothelial cells (Prox1^+^Lyve1^+^) and matrix-regulating endothelial cells as distinct subsets [36], though their significance in ER stress regulation awaits further exploration.

### 6.3. Chapter 6 Summary

Unlike other cell types, the core feature of ER stress in endothelial cells is its hemodynamic dependence. Laminar flow maintains low ER stress, while disturbed flow activates ER stress and drives dysfunction [54]. At the single-cell level, classical endothelial cells exhibit UPR downregulation in advanced AS, whereas the ER stress features of specialized subsets such as ACKR1^+^ and PLVAP^+^ require further investigation. Overall, research on endothelial ER stress remains in its early stages, and more single-cell-level functional validation is needed in the future.

## 7. Targeting ER Stress in Atherosclerosis: From Global to Precision

### 7.1. From Pan-ER Stress Modulators to Subset-Specific Precision Strategies

Several compounds have been reported to modulate ER stress, among which the chemical chaperones 4-PBA and TUDCA are the most widely studied. In animal models, 4-PBA treatment can alleviate endoplasmic reticulum stress, reduce macrophage apoptosis, and stabilize plaques [55]. STF-083010, an IRE1α inhibitor, can alleviate atherosclerotic lesions in mice [56,57]. However, these drugs lack cell type specificity. They may inhibit pathological endoplasmic reticulum stress in monocytes, while also interfering with the protective UPR signaling pathways in M2-like macrophages or endothelial cells. This limitation highlights the necessity of developing more precise intervention treatments.

When the PERKeIF2αCHOP pathway is continuously activated in foam macrophages, it promotes apoptotic cell death and secondary necrosis, thereby expanding the necrotic core. This is also a hallmark of vulnerable plaques [58,59]. In advanced lesions, the apparent attenuation of M2-like macrophage UPR markers may reduce the efferocytic ability and exacerbate the accumulation of apoptotic debris [60]. In SMCs, the duration and intensity of ER stress determine whether the outcome is adaptive recovery or maladaptive phenotypic switching [61]. As a result, the same ER stress signals may be due to the role of cell types and UPR branches and produce radically different plaque compositional outcomes.

We have recognized that endoplasmic reticulum stress plays different roles in different cell subpopulations, so the treatment strategies should be adjusted accordingly. In monocytes, we can inhibit their pro-inflammatory differentiation by suppressing the PERK-eIF2α-ATF4-CHOP signaling pathway. In Trem2^+^ macrophages, we can reduce foam cell formation by interfering with the IRE1α-XBP1 pathway [25]. In M2-like macrophages, we need to restore the unfolded protein response ability by enhancing the expression or function of HSPA5 [62,63]. And in endothelial cells, moderately activating the ATF6 pathway helps restore barrier function [46,64]. In SMCs, where ER stress exerts both pro-apoptotic and adaptive protective effects, direct intervention carries considerable risk; a more rational approach is to target downstream transcription factors, such as inhibiting Klf4 to block pro-inflammatory and osteogenic switching [38,65,66], or enhancing TCF21 to promote fibrous cap stabilization [9].

In order to translate these specific strategies of these subgroups into clinical practice, diagnostic tools also need to progress simultaneously. Currently, no clinically approved diagnostic directly profiles cell-type-specific ER stress in patients. However, several promising approaches are emerging: (i) blood-based biomarkers, such as sTREM2 and NLRP3, which correlate with ER stress-related gene signatures in advanced plaques [67,68]; (ii) folate receptor (FOLR2)-targeted PET/SPECT imaging, which exploits the high expression of FOLR2 on activated plaque macrophages to visualize inflammatory burden [69]; and (iii) single-cell RNA sequencing of circulating monocytes or plaque specimens from carotid endarterectomy, which can resolve the ER stress activation patterns across different cell subsets [70]. These tools, though still largely investigational, may eventually enable the identification of patients with specific cell-type ER stress activation patterns.

Yet diagnosis is only the first step, therapy also requires precision delivery. Most of these strategies currently remain at the conceptual level. A major obstacle is the lack of delivery systems capable of distinguishing different cell types. Nanoparticle carriers and gene tools driven by cell type-specific promoters represent possible directions for addressing this challenge [71]. Despite many hurdles, a conceptual clinical pathway can be envisioned as a stepwise process: initial screening and confirmation using the biomarker and imaging tools described above, followed by precise subtyping by single-cell analysis when clinically accessible [70], leading to the selection of potential subset-appropriate ER stress modulators, with longitudinal monitoring of treatment response through serial imaging and biomarker tracking [68]. This framework is currently still in the speculative stage. We should recognize that these steps may involve iterative feedback in practice, rather than a strictly linear sequence. Nonetheless, this framework outlines a possible translational roadmap for future refinement and validation.

### 7.2. Emerging Strategies

The conceptual pathway outlined above provides a framework for clinical translation, but its realization depends on the availability of experimentally validated interventions to populate each step.

How can true subset-specific targeting be achieved? One approach is to bypass systemic ER stress modulation altogether. The chemical chaperone 4-PBA can reprogram monocytes into an immune-resolving state, and adoptive transfer of these cells alleviates atherosclerosis [72]. Another direction is to achieve cell type-specific delivery. The pH-responsive nanomedicine mPEG@ELA-11 exploits the acidic microenvironment of plaques for targeted release and modulates macrophage function through AKT-ER stress pathway inhibition [73]. Pathway-specific inhibitors also offer precision at the molecular level. PERK inhibitor ISRIB ameliorates vascular calcification [74], and IRE1 inhibitors reduce plaque macrophage numbers and increase collagen deposition [56]. A third, more indirect route is to regulate upstream signals with built-in cell type preference. Meteorin-like protein (METRNL) selectively inhibits foam cell formation originating from vascular smooth muscle cells by enhancing the KIT signaling pathway and alleviating endoplasmic reticulum stress [75]. These strategies are currently mainly at the preclinical stage, but they illustrate the diversity of cell-based, nanomedicine-based, pathway-specific, and upstream regulatory approaches being explored to realize subset-specific ER stress modulation.

### 7.3. Chapter 7 Summary

Therapeutic intervention targeting ER stress in atherosclerosis remains in its early stages. Although progress has been made in developing diagnostic tools and conceptual frameworks, cell type-specific delivery remains a major obstacle to clinical translation. The differential response model proposed in this review provides a theoretical basis for the next generation of therapeutic strategies, but achieving true subgroup-specific regulation remains a long-term goal. It is comforting to note that marketed cardiovascular drugs (such as metformin, empagliflozin and liraglutide) have been proven to regulate endoplasmic reticulum stress [76,77,78], indicating pharmacological modulability in this direction. However, these agents lack cell type specificity, and substantial gaps remain before precision ER stress-targeted therapies can be realized in clinical practice.

## 8. Discussion

The traditional view holds that endoplasmic reticulum stress (ER stress) functions as a pro-atherosclerotic signal, driving inflammatory responses in macrophages, apoptosis in smooth muscle cells (SMCs), and endothelial cell dysfunction [3,4,5]. However, when we re-examine this view from the single-cell perspective, we will find that the reality is much more complex [79].

Endoplasmic reticulum stress shows directional differences among different cell subpopulations. In monocytes and Trem2^hi^ type macrophages, endoplasmic reticulum stress is activated and is associated with pro-inflammatory differentiation and foam cell formation. However, in M2-like macrophages and endothelial cells derived from advanced atherosclerotic plaques, the markers of endoplasmic reticulum stress and the core unfolded protein response (UPR) gene levels are instead decreased. This asymmetry indicates that endoplasmic reticulum stress is not a uniform pathological signal but rather there are differences in its interpretation by different cells.

This difference not only determines the composition of macrophages in plaques but also has important significance for prognosis and targeted therapy. During the formation of atherosclerotic plaques, the composition of macrophages shows a continuous trajectory from reparative to pathogenic. Lyve1^+^ tissue-resident macrophages, which co-express TimD4 and folate receptor 2 (FOLR2), are relatively enriched in the initial stages of the lesion and are involved in lipid clearance and tissue homeostasis [7,60]. With the progression of the lesion, the plaque microenvironment gradually changed to a pro-inflammatory and lipid-rich state, and Trem2^hi^ and SPP1^+^ foam macrophages gradually increased to the dominant subsets. This phenotypic change may be partly due to the differential sensitivity of different macrophage subpopulations to endoplasmic reticulum stress. Trem2-deficient foam macrophages exhibit an enhanced ER stress response under lipid load, while macrophages from symptomatic plaques show higher ER stress levels through PERK pathway activation [11,80]. Entered the late stage of unstable plaque, IL1b^+^ inflammatory macrophages number increased significantly, and has the repair function significantly reduce the number of sample M2 macrophages [12,60]. The reduction in endoplasmic reticulum stress markers in the latter may weaken its repair function, including support for cell burial, which is highly dependent on MerTK. Its dysfunction in advanced plaques is further exacerbated by the upregulation of CD47/SIRPα and the impairment of bridging molecular signals [60,81]. Thus, the progressive shift in macrophage composition reflects the impact of ER stress heterogeneity on plaque stability. This compositional change not only drives plaque progression but also provides the basis for prognostic evaluation and targeted therapeutic intervention.

The prognostic value of macrophage subpopulation markers likely reflects ER stress burden within the plaque microenvironment. This is supported by evidence at multiple levels. At the tissue level, SPP1^+^/VCAN^+^, IL1B^+^, FLT3LG^+^ macrophages and lipid-associated macrophages (LAMs, marked by PLIN2 and TREM1) are associated with increased plaque immune infiltration and post-operative MACE risk [82,83]. TREM1 expression is directly regulated by ER stress [84], and ER stress-related gene signatures in advanced plaques correlate with M0 and M2 macrophage infiltration [82,85]. At the serum level, sTREM2 is a specific prognostic biomarker, all-cause mortality HR 2.37 for the highest quartile [86], and TREM2 itself is functionally linked to ER stress signaling [87]. IL-1β is also an independent predictor, with its upstream NLRP3 inflammasome regulated by the PERK-eIF2α-CHOP pathway. At the systemic level, the peripheral M1/M2 ratio predicts post-PCI microvascular dysfunction and heart failure after ischemia–reperfusion injury [88,89]. This polarization balance is itself influenced by ER stress, as PERK-eIF2α activation drives M1 polarization while UPR inhibition may weaken M2 repair functions. Together, these findings indicate that macrophage subset markers reflect not only cell identity but also the underlying ER stress burden driving plaque progression.

The heterogeneity of macrophage subpopulations also provides an entry point for precise treatment, and the cell type-specific distribution of endoplasmic reticulum stress enables these strategies to be more targeted. FOLR2 expression on Lyve1^+^ resident macrophages enables folate-conjugated nanocarriers to deliver ER stress-modulating agents selectively to the most stressed subsets [90,91]. Anti-CD47 nanoparticles targeting the CD47-SIRPα axis restore efferocytosis and reduce plaque burden [92], counteracting the efferocytic dysfunction that results from insufficient ER stress tolerance in M2-like macrophages. Thus, macrophage subset-based prognosis and therapy converge on a common principle: ER stress is concentrated in specific pathogenic subsets, and targeting these subsets may improve clinical outcomes in atherosclerosis.

Lipid-lowering therapies, including statins and PCSK9 inhibitors, which are one of the core strategies for the treatment of cardiovascular diseases, exert anti-atherosclerotic effects not only by reducing circulating cholesterol but also by directly modulating macrophage subset composition. Statins promote M1-to-M2 polarization, enhance CCR7-dependent macrophage emigration, and suppress foam cell formation via PI3K/Akt/mTOR inhibition and ABCA1/G1 upregulation [93,94]. PCSK9 inhibitors block PCSK9-mediated TLR4-NF-κB-NLRP3 and LOX-1 signaling, thereby inhibiting M1 polarization and enhancing efferocytosis [95,96].

The results of the SMC study reveal that the activation of the unfolded protein response (UPR) itself is not pathological, and its ultimate outcome depends on how cells interpret this signal. In SMCs, mild and persistent endoplasmic reticulum stress seems to be compatible with the recovery of contraction markers, while severe or long-term stress triggers cell apoptosis and phenotypic transformation [53,97]. This suggests that there may be a threshold effect, beyond which the adaptive UPR mechanism will transform into a pathological state. The specific factors determining this threshold are not clear, but they may involve the duration of stress, the specific UPR pathways involved, and the initial state of the cells. Studies have found that specific inhibition of PERK in smooth muscle cells can reduce plaque formation [98], while another study indicates that PERK is not essential for phenotypic transformation [99]. These results further suggest that different UPR pathways may have overlapping or context-dependent functions. To clarify this issue, systematic comparisons of the activity of different UPR pathways under different stress durations and in different cell environments are still needed.

The conflicting evidence regarding PERK signaling in SMCs warrants critical reconciliation. On one hand, SMC-specific Perk deletion reduces plaque formation by approximately 80% [98]; on the other, Perk has been reported to be dispensable for SMC phenotype switching [99]. We propose that this apparent contradiction reflects differences in the experimental contexts rather than a true biological discrepancy. In hypercholesterolemic models, where cholesterol directly activates the PERK-eIF2α-ATF4-KLF4 axis [100], PERK is a critical driver of SMC de-differentiation and migration [98,101]. Conversely, in settings where cholesterol loading is not the primary driver or where alternative UPR pathways such as IRE1α or ATF6 can compensate, PERK may become dispensable [61,99]. Furthermore, PERK appears to play a stage-specific role. It is critical during the early migration of SMCs into the intima, but less so for maintaining the fully modulated phenotype in advanced lesions, where UPR activity is low [99]. In summary, rather than being mutually exclusive, these findings collectively illustrate the plasticity of the SMC stress response. PERK is a key driver under hypercholesterolemic stress, but its necessity is contextually overridden by other pathways when the stressor or disease stage differs.

The differences in endoplasmic reticulum stress responses within endothelial cells are mainly determined by their physical location. In regions prone to the formation of atherosclerosis, endothelial cells experience blood flow disorders and exhibit higher endoplasmic reticulum stress activity than those in areas protected against atherosclerosis [102]. This hemodynamic effect is further regulated by the extracellular matrix. Endoplasmic reticulum stress is selectively induced only in cells attached to fibronectin but does not occur in cells attached to basement membrane proteins. And this effect depends on the integrin signaling pathway [103]. These observations indicate that endoplasmic reticulum stress in endothelial cells is influenced by both the blood flow pattern and the matrix composition, thereby adding a spatial dimension to the heterogeneity.

Beyond hemodynamic and matrix regulation, ER stress also influences endothelial cell function through the induction of endothelial-to-mesenchymal transition (EndMT), a cellular program in which endothelial cells acquire mesenchymal-like phenotypes, leading to enhanced migration, extracellular matrix production, and pro-inflammatory activation. ER stress has been identified as a critical upstream driver of EndMT. For instance, the gut microbiota metabolite trimethylamine N-oxide (TMAO) promotes EndMT and atherosclerosis through activation of the PERK-eIF2α pathway [104], while disruption of the circadian clock gene exacerbates ER stress-induced EndMT via the IRE1α-XBP1 axis [105]. These observations suggest that ER stress may contribute to endothelial dysfunction and plaque vulnerability, at least in part, through the promotion of EndMT. Having discussed the specific mechanisms in endothelial cells, we now turn to a broader question: why do different cell types exhibit opposing directions of UPR regulation?

**Figure 3:**
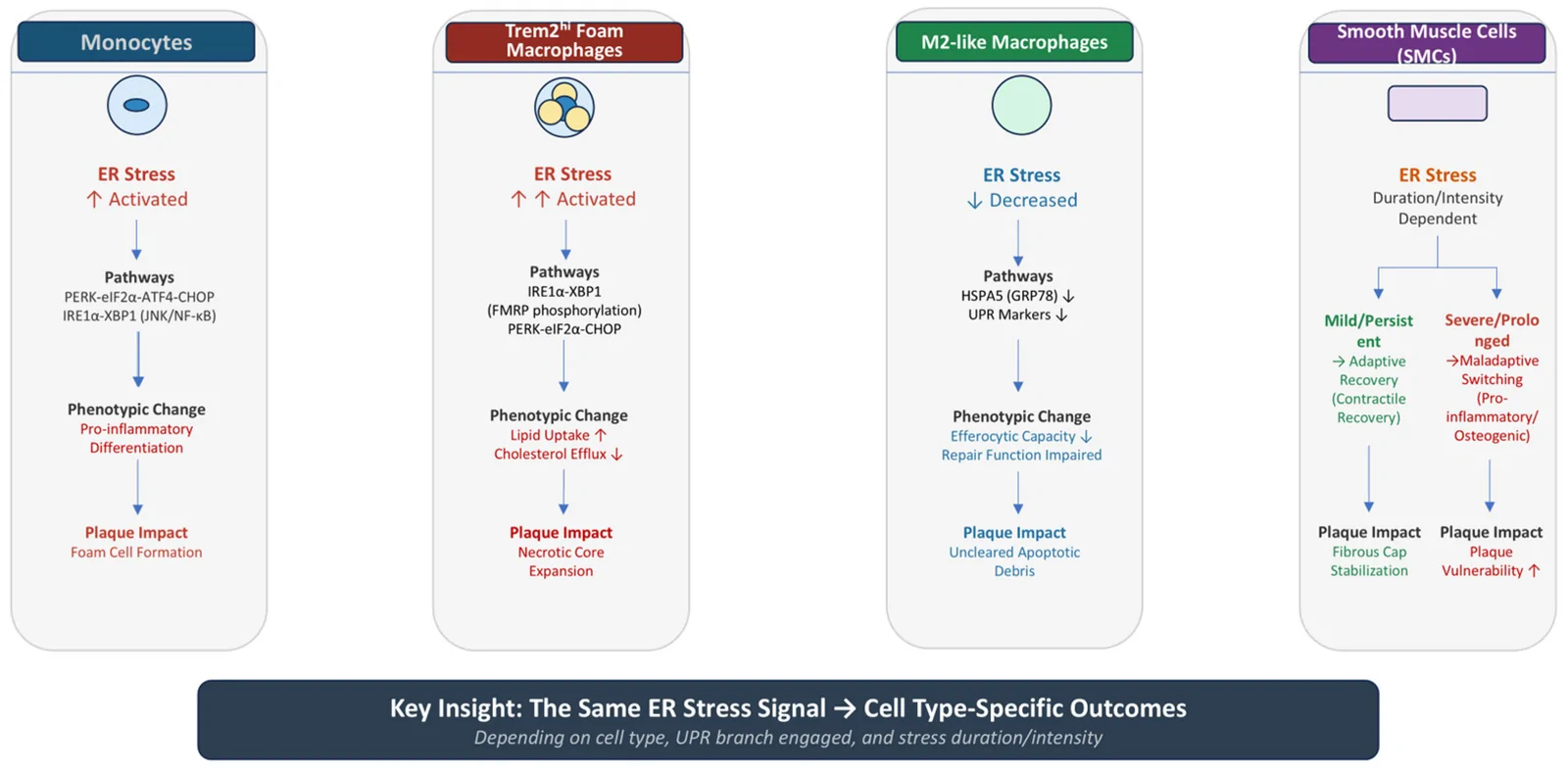
Cell type-specific ER stress patterns and phenotypic changes in atherogenesis. This figure provides a comparative overview of ER stress activation patterns across different cell types involved in atherosclerosis and their associated phenotypic changes. Arrowheads in the signaling pathways indicate the direction of signal transduction; upward (↑) and downward (↓) arrows denote increased or decreased expression or activity, respectively. Monocytes entering the plaque microenvironment exhibit activated ER stress through PERK-eIF2α-ATF4-CHOP and IRE1α-XBP1 pathways, driving pro-inflammatory differentiation and subsequent foam cell formation. Trem2^hi^ foam macrophages display the most pronounced ER stress activation, primarily via IRE1α-XBP1-mediated FMRP phosphorylation and PERK-eIF2α-CHOP, which promote lipid uptake, inhibit cholesterol efflux, and accelerate necrotic core expansion. In contrast, M2-like macrophages from advanced plaques show decreased UPR markers (e.g., HSPA5/GRP78), which may compromise their efferocytic capacity and repair functions, leading to accumulation of uncleared apoptotic debris. Smooth muscle cells (SMCs) exhibit a unique, duration- and intensity-dependent response: mild and persistent ER stress permits adaptive recovery and maintenance of contractile markers, whereas severe or prolonged stress triggers maladaptive phenotypic switching toward pro-inflammatory and osteogenic phenotypes. Thus, the same ER stress signal yields fundamentally different cellular outcomes depending on cell type, UPR branch engaged, and stress duration/intensity.

As mentioned above, we believe that the UPR directional differences, namely, monocytes and Trem2^hi^ rise in macrophages and endothelial cells and cut sample M2 macrophages. This raises a question: why do only certain cell types adopt the transcriptional inhibition of the endoplasmic reticulum stress sensor? This divergence reflects cell type-specific differences in stress duration, cellular turnover, metabolic state, and functional vulnerability. Monocytes and Trem2^hi^ macrophages are relatively short-lived cells constantly replenished from the circulation. Their UPR activation represents an acute response to lipid loading and inflammatory stimuli, driving foam cell formation and pro-inflammatory polarization [58,61]. In contrast, endothelial cells are long-lived residents chronically exposed to disturbed flow, oxidative stress, and metabolic insults over extended periods [106]. Under such persistent stress, they may actively downregulate UPR sensors as a “stress adaptation” or “exhaustion” strategy to avoid chronic inflammation [107]. For M2-like macrophages, the reduced UPR markers may reflect different metabolic programs. They rely more on oxidative phosphorylation but have lower requirements for endoplasmic reticulum protein synthesis or represent a chronic stress state that impairs the function of inflammatory cells [81,108]. Thus, the directionality of UPR regulation is not random, different cell subpopulations interpret endoplasmic reticulum stress differently. The cell type-specific ER stress patterns and phenotypic changes discussed above are summarized in Figure 3.

It should be noted, however, that most of the single-cell evidence discussed in this article is derived from cross-sectional analyses of late-stage human plaques. Therefore, the temporal dynamic changes in ER stress during plaque formation and progression mainly rely on pseudo-time reconstruction techniques for inference rather than direct observation. While pseudotime analysis provides a valuable hypothesis-generating framework, it cannot substitute for true longitudinal data, and the inferred trajectories should be interpreted as hypothetical rather than definitive. The functional impacts caused by the weakened endoplasmic reticulum stress in M2-like macrophages and endothelial cells are still unclear. The proposed therapeutic hypothesis in this article needs to be verified in specific cell type experimental models.

## 9. Conclusions

Single-cell RNA sequencing technology has reshaped our understanding of the function of endoplasmic reticulum stress in atherosclerosis. Endoplasmic reticulum stress is not a single signal promoting atherosclerosis but rather functions as a regulatory factor in the plaque microenvironment, being context-dependent and cell type-specific. The role of endoplasmic reticulum stress varies among different cell types. The same signal can produce completely different biological effects depending on the cell type and specific environment. It is activated in monocytes and Trem2^hi^ macrophages, triggering an inflammatory response and promoting foam cell formation; while in M2-like macrophages and endothelial cells within advanced plaques, the level of endoplasmic reticulum stress is reduced, and the expression of core unfolded protein response (UPR) genes is also downregulated; in smooth muscle cells, endoplasmic reticulum stress can lead to phenotypic transformation, and mild UPR activation may maintain contractile homeostasis.

This mechanistic insight has begun to translate into clinical perspectives. Specific macrophage subpopulations identified through single-cell analysis, such as SPP1/VCAN^+^ and lipid-associated macrophages, have shown prognostic value for cardiovascular outcomes. Their association with endoplasmic reticulum stress characteristics indicates that these markers may indirectly reflect the endoplasmic reticulum stress load within the plaque. Moreover, the cell type-specific distribution of ER stress opens avenues for precision intervention, as surface markers like FOLR2 on resident macrophages and CD47 on apoptotic cells can be exploited for targeted delivery or enhanced efferocytosis.

This insight was achieved thanks to the application of single-cell sequencing technology. Without the high-resolution data provided by scRNA-seq, we would not be able to distinguish Lyve1-positive macrophages from Trem2^+^ macrophages, nor could we distinguish contracting smooth muscle cells from synthetic smooth muscle cells, and these distinctions are the basis of the differential patterns described in this paper. Recognizing this heterogeneity is the first step in understanding the mechanism of endoplasmic reticulum stress in atherosclerosis.

## Figures and Tables

**Table 1:** Cell type-specific ER stress responses in atherosclerosis.

Cell Type	Subsets & Key TFs	Key Pathways	Outcomes
Monocyte	CD14^+^FCN1^+^	PERK-CHOP; IRE1α-XBP1	Inflammation; pyroptosis
Trem2^hi^ Macrophage	Trem2^hi^SPP1^+^ (foam-like); Il1b^+^ (inflammatory)	PERK-CHOP; IRE1α-XBP1; NLRP3	Foam cell formation; apoptosis; impaired efferocytosis
M2-like Macrophage	CD163^+^MRC1^+^; HSPA5 low	UPR signaling (relative); chaperone insufficiency	Reparative function?
SMC	Contractile (ACTA2^+^); Synthetic (Klf4^+^); Fibromyocyte (TCF21^+^)	PERK-eIF2α-ATF4; IRE1α-XBP1	Phenotypic switching; calcification; adaptive UPR
Endothelial Cell	Classical (PECAM1^+^); ACKR1^+^; Prox1^+^ (lymphatic)	IRE1α-XBP1; ATF6	Adhesion; apoptosis; barrier dysfunction

Notes: Subsets and transcription factors (TFs) are derived from scRNA-seq studies. “HSPA5 low” indicates reduced chaperone expression. Question mark (?) denotes functional consequence not yet established. Full mechanistic details are discussed in the main text.

## Data Availability

No new data were created or analyzed in this study. Data sharing is not applicable to this article.

## Abbreviations

The following abbreviations are used in this manuscript:

AS	Atherosclerosis
ER stress	Endoplasmic Reticulum Stress
UPR	Unfolded protein response
ERAD	ER-associated degradation
Sc-RNA seq	Single-cell sequencing
VSMCs	Vascular smooth muscle cells
SMC	Smooth muscle cells
VLDL	Very low-density lipoprotein
CCN2	Cellular communication network factor 2
FMRP	Fragile X mental retardation protein
CAD	Coronary artery disease
NOX	NADPH oxidase
SR-A	Scavenger receptor A
ROS	Reactive oxygen species
4-PBA	4-Phenylbutyric Acid
FOLR2	folate receptor
EndMT	endothelial-to-mesenchymal transition
TMAO	trimethylamine N-oxide
